# The Tumor Microenvironment and Cancer

**DOI:** 10.1155/2014/573947

**Published:** 2014-12-22

**Authors:** Zhen Chen, Zhiqiang Meng, Lijun Jia, Rutao Cui

**Affiliations:** ^1^Department of Integrative Oncology, Fudan University Shanghai Cancer Center, Shanghai 200032, China; ^2^Department of Oncology, Shanghai Medical College, Fudan University, Shanghai 200032, China; ^3^Cancer Institute, Fudan University Shanghai Cancer Center, Shanghai 200032, China; ^4^Department of Dermatology, Boston University School of Medicine, Boston, MA 02118, USA

Tumors are now recognized as structures of multiple cell types. It is increasingly appreciated that as the cancer progresses, the surrounding microenvironment is activated in support, coevolving through continuous paracrine communication and supporting carcinogenesis. Therefore, the characterization of these interactions, and the molecular identification of key mediators, will provide insights into tumor biology and suggest further novel therapeutic options.

In this special issue, C. Roma-Rodrigues et al. reviewed the role of exosome in tumor microenvironment, especially the cancer derived exosomes in the development and progression of cancer through modulation of intercellular communication within the tumor microenvironment by the transfer of protein, lipid, and RNA cargo. S. L. Schlereth et al. reviewed the crosstalk between cancer cells and tumor microenvironment that involved in the process of lymphangiogenesis. They showed that lymphangiogenesis is regulated by cells of the tumor microenvironment, including cancer-associated fibroblasts, mesenchymal stem cells, dendritic cells, or macrophages. Therefore, targeting prolymphangiogenic tumor microenvironment may represent a promising therapeutic option for cancer metastasis. J. Zhou et al. reviewed that chemoattractant receptors, a family of seven transmembrane G protein coupled receptors (GPCRs) combined with their ligands, are involved in almost every step of tumor development and progression such as increasing tumor cell migration, invasion, and metastasis. Bone metastases remain as the major problem for cancer patients. Bone metastases account for decreased quality of life and ultimately death of prostate, breast, and lung cancer patients. In this issue, D. Buenrostro et al. reviewed that the bone microenvironment cells including the fibroblasts, osteoblasts, osteoclasts, immune cells, and others may interact with the tumor cells and promote tumor cells to metastasize to the bone and other sites. While A. Romano et al.'s review focused on the role of immunological dysregulation in multiple myeloma progression and their potential clinical implications as novel therapeutic target.

We hope that this issue will help researchers understand the interactions between tumor cells and tumor microenvironment that mediate tumor progression and metastasis. We also hope that this special issue will initiate new discussions relating to the development of novel anticancer therapies or preventive strategies based on an understanding of the communication within the tumor microenvironment.

*Zhen Chen* *Zhiqiang Meng* *Lijun Jia* *Rutao Cui*

